# Bronchoscopic management of bronchopleural fistula using free fat pad transplant with platelet-rich plasma: a case study

**DOI:** 10.1186/s13019-024-02900-x

**Published:** 2024-06-25

**Authors:** Yu-Hsiang Wang, Hsu-Chih Huang, Frank Cheau-Feng Lin

**Affiliations:** 1 Department of Surgery, Chung Shan Medical University Hospital, Taichung City, Taiwan; 2 Division of thoracic surgery depart, Chung Shan Medical University Hospital, 402 No. 110, Section 1, Jianguo North Road, Taichung City, Taiwan; 3 Chung Shan Medical University, Taichung City, Taiwan

## Abstract

**Background:**

A bronchopleural fistula (BPF) occurs when an abnormal connection forms between the bronchial tubes and pleural cavity, often due to surgery, infection, trauma, radiation, or chemotherapy. The outcomes of both surgical and bronchoscopic treatments frequently prove to be unsatisfactory.

**Case presentation:**

Here, we report a case of successful bronchoscopic free fat pad transplantation combined with platelet-rich plasma, effectively addressing a post-lobectomy BPF. Contrast-enhanced chest tomography revealed pleural thickening with heterogeneous consolidations over the right upper and middle lobes, indicative of destructive lung damage and bronchiectasis. The patient underwent thoracoscopic bilobectomy of the lungs. During surgery, severe adhesions and calcification of the chest wall and lung parenchyma were observed. The entire hilar structure was calcified, presenting challenges for dissection, despite the assistance of energy devices. Bronchoscopic intervention was required, during which two abdominal subcutaneous fat pads were retrieved.

**Conclusion:**

This innovative approach offers promise in the management of BPF and signals potential advancements in enhancing treatment efficacy and patient recovery.

## Background

Bronchopleural fistula (BPF), a major complication of lung resection surgery, is associated with a high mortality rate. The presence of a fistula results in persistent air leakage through the stump, leading to the failure of lung expansion and recurrent pneumonia. Effective BPF treatments include surgical and bronchoscopic interventions. Bronchoscopic interventions, the bronchoscopic placement of blocking agents, have emerged as an alternative therapy to treat persistent air leaks and are particularly valuable for patients that are unsuitable for surgical intervention. Several blocking agents have been proposed for ventricular septal defects (VSDs), including fat pads, airway stents, endobronchial valves, and occluders. However, a consensus on their effectiveness and long-term outcomes remains elusive. In this report, we present a case of bronchoscopic free fat pad transplantation with platelet-rich plasma for the treatment of a post-lobectomy BPF .

## Case presentation

A 39-year-old man, who worked as a tunnel worker for a decade, was diagnosed with bilateral pulmonary tuberculosis and lung destruction. Unfortunately, owing to work constraints, the patient received only partial treatment. Years later, he visited Chung Shan Medical University Hospital and presented with hemoptysis. Two years prior, he underwent transcatheter arterial embolization for severe hemoptysis. However, the intermittent hemoptysis persisted, leading to several admissions during which he received conservative treatment.

**Fig. 1:**
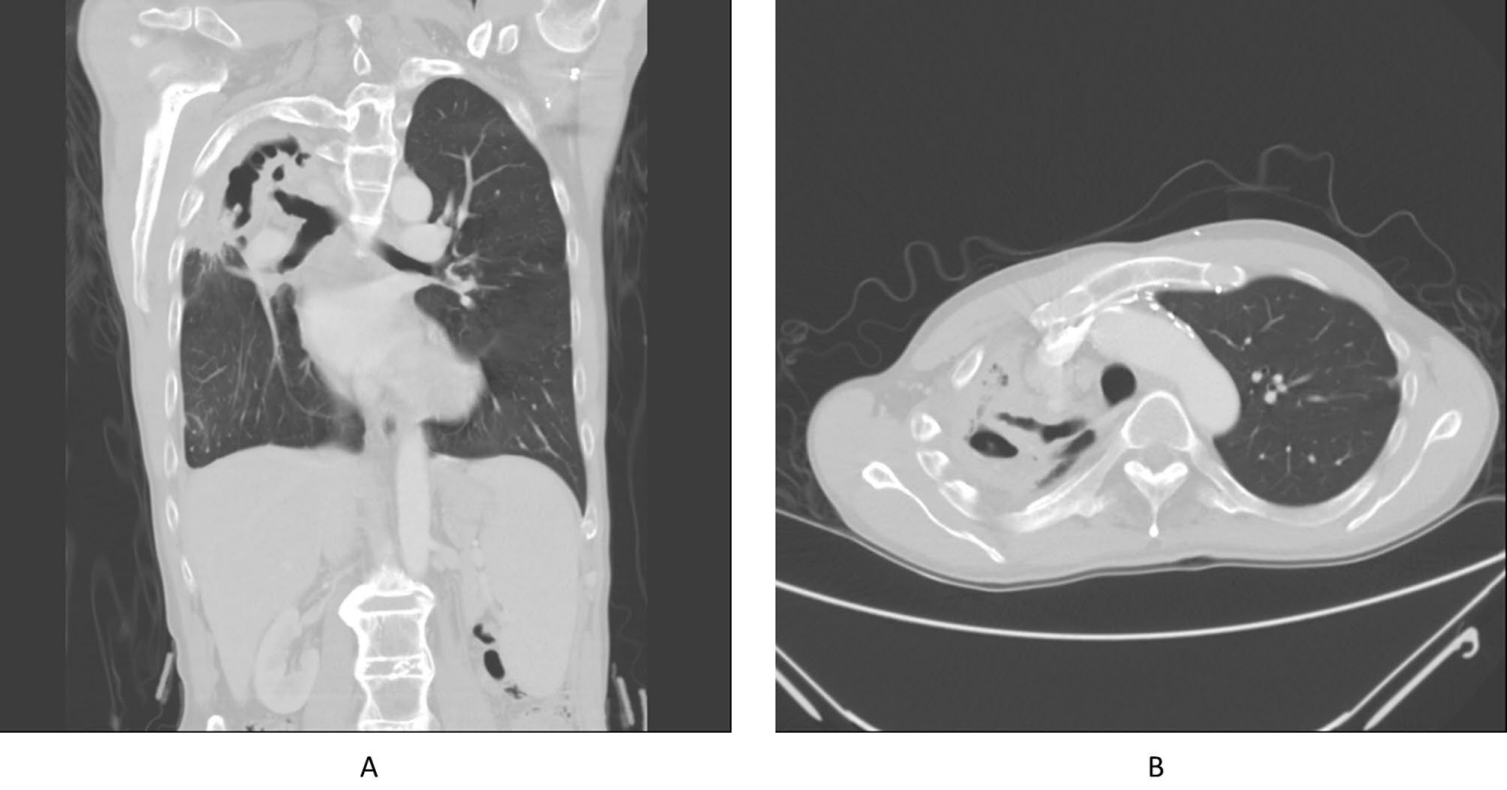
**A-B**: Computed tomography (CT) scan of patient : The CT scan reveals a large consolidation in the right upper lobe with some calcification observed around the bronchial region, particularly in the upper lobe bronchus

Contrast-enhanced chest tomography revealed pleural thickening with heterogeneous consolidations over the right upper and middle lobes, indicative of destructive lung damage and bronchiectasis (Fig. 1A-B). His preoperative lung function showed poor forced expiratory volume in one second (FEV1 = 47% prediction) and forced vital capacity (FVC = 36% prediction), consistent with obstructive lung disease. Given the recurrent hemoptysis and failure of conservative treatment, lobectomies of the right upper and middle lobes were recommended.

The patient underwent thoracoscopic bilobectomy of the lungs. During surgery, severe adhesions and calcification of the chest wall and lung parenchyma were observed. The entire hilar structure was calcified, presenting challenges for dissection, despite the assistance of energy devices. Identification of the tortuous pulmonary artery and bronchus led to the division of the right upper and middle lobes, using endovascular gastrointestinal anastomosis stapler (Endo GIA; Medtronic, Minneapolis, MN, USA) and linear stapler (DST Series™ TA™ 60 mm, Medtronic, Minneapolis, MN, USA) assist.

Postoperatively, small air leaks developed through the parenchyma and stump side that required repair using 6 − 0 PDS sutures. Unfortunately, one week later, the patient’s chest tube revealed a worsened air leak, and a follow-up film indicated severe right lower lobe collapse, indicative of BPF formation. A second surgical procedure revealed a fistula, measuring 8 mm, over the right upper lobe stump. An intercostal muscle flap was created to repair the BPF; however, large volume air leakage persisted.

**Fig. 2:**
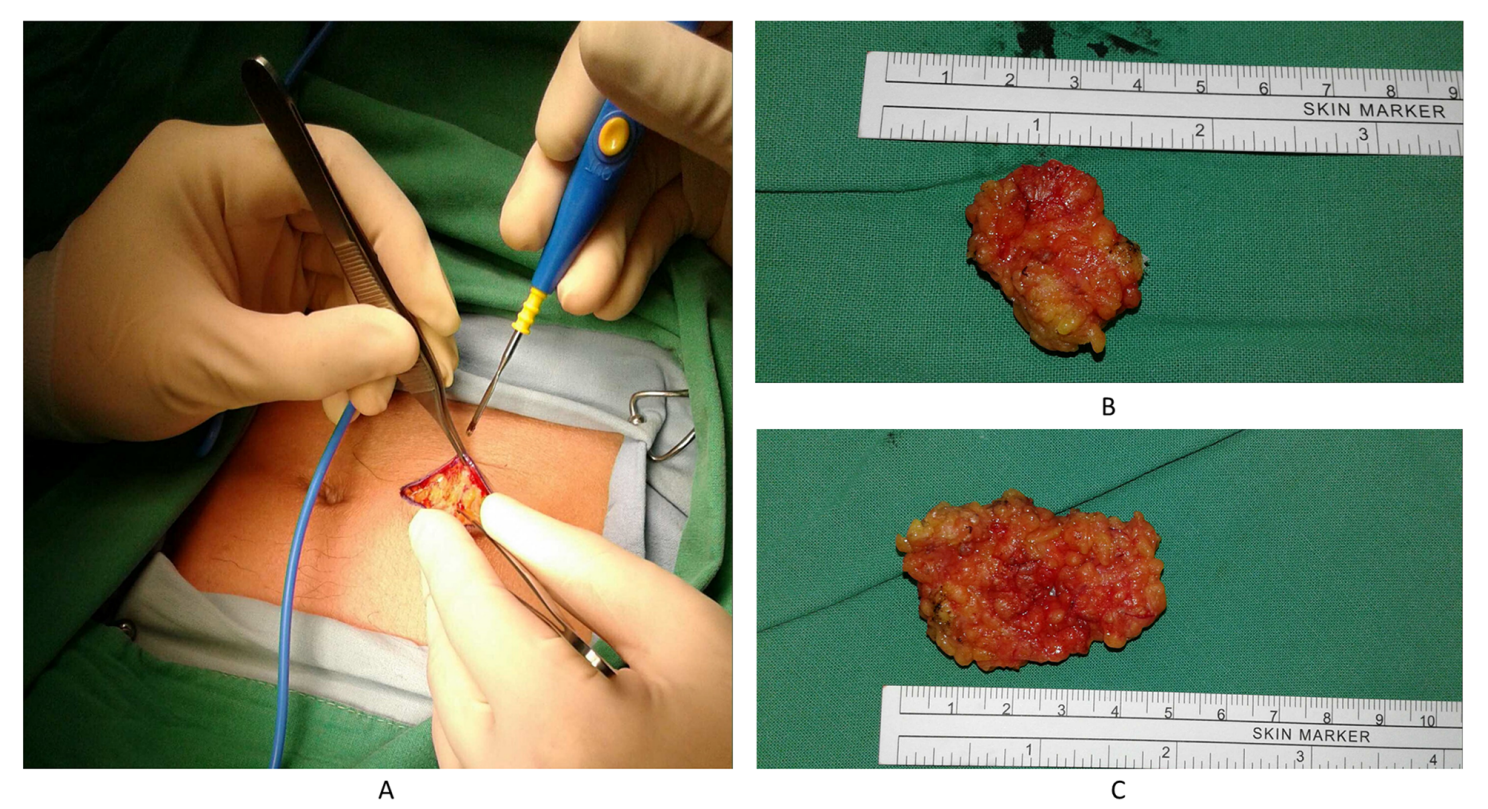
**A**: Periumbilical incision and exposure of the abdominal fat pad. **B**: Harvest of two fat pads: one measuring 3*2 cm and the other 4*2 cm in size

**Fig. 3:**
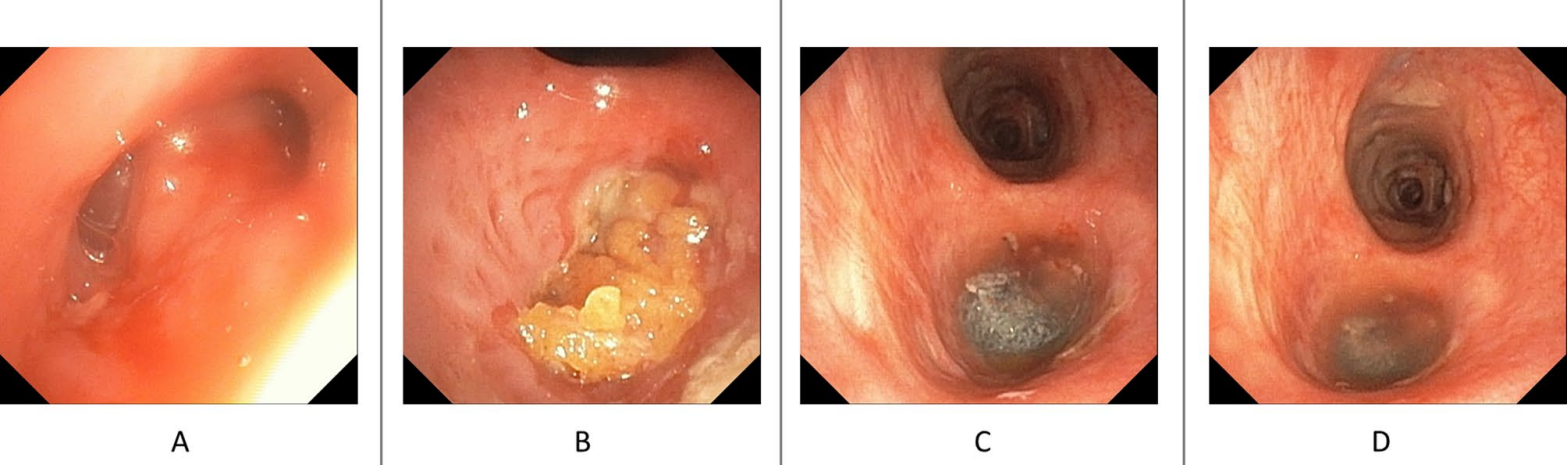
Fat pad retrieval of bronchopleural fistula treatment. **A**: The previous TA stump formation and the bronchial pleura fistula, the fistula size is about 0.8 cm in size. **B**: Two fat pad were placed in layers to filled the whole upper lobe bronchus. **C**: The Neoviel was cut into small piece and filled up the fat pad. **D**: The PRP was finally filled into the fat pad and Neoviel to stabilized and nourished the structure

**Fig. 4:**
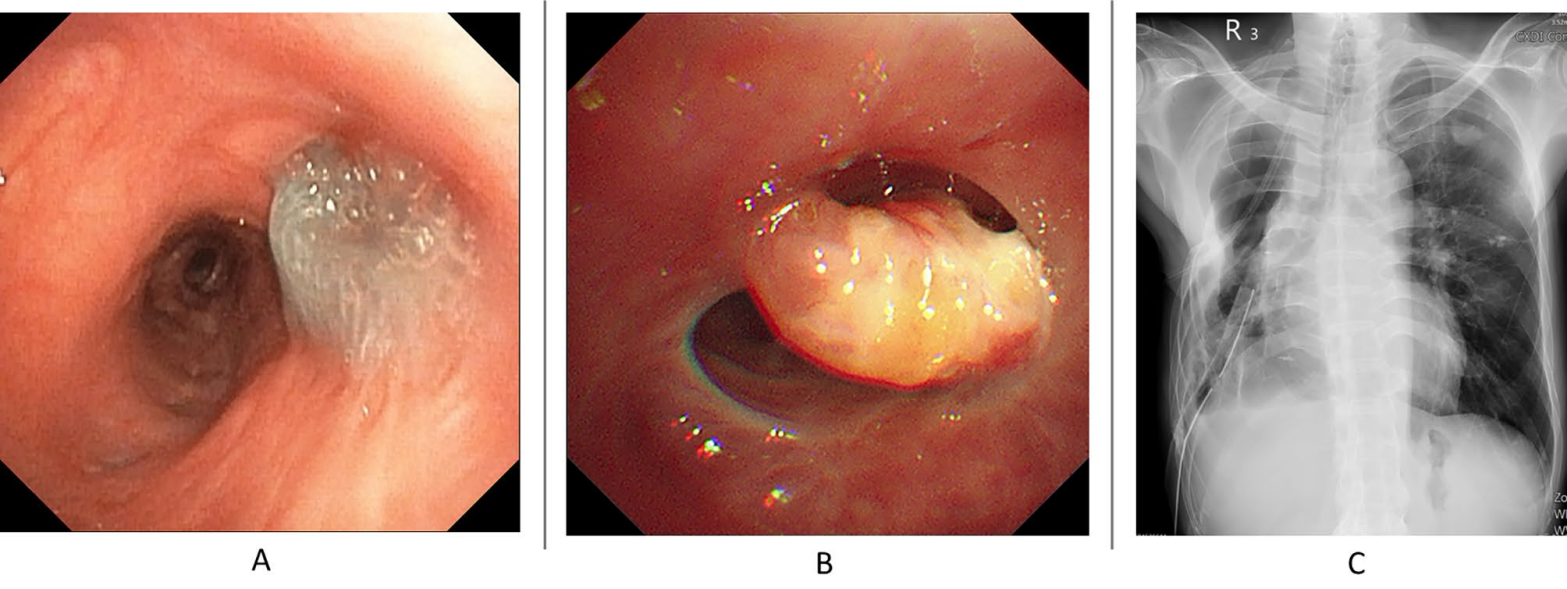
**A**: On postoperative day 7, the PRP/Neoviel/Fat pad complex remains firm with no signs of shrinkage. **B**: By postoperative day 60, the Neoviel is resolving, and the fat pad has shrunk by approximately 50%. No recurrence of fistula is observed. **C**: Although there is still dead space over the previous right upper lobectomy site, lung expansion improves under chest tube drainage

To address the issue, two abdominal subcutaneous fat pads were retrieved for treatment (Fig. 2A-B). The patient was positioned in the right decubitus position, and the was noted over the apical segment of the right upper lobe (8 mm in size, Fig. 3A). The bronchial stumps were removed using electrocauterization. The obtained subcutaneous fat pads were applied to the lesion and covered with platelet-rich plasma (PRP) and Neoveil™ (Gunze Ltd., Japan) (Fig. 3B-D). Following surgery, sedation was maintained, and the right decubitus position was maintained for three days. Follow-up bronchoscopy on (7-days post-surgery) revealed that the fat pad was firm and steady with no fistula formation (Fig. 4A). The patient was weaned off his ventilator on Day 15 post-surgery and later transferred to the general ward. Follow-up bronchoscopy (60-days post-surgery) revealed shrinkage of the fat pad and resolution of the outside Neoveil coverage with no fistula formation (Fig. 4B). Although a follow-up video recording revealed a persistent dead space over the right chest wall, considering the relatively stable condition, the patient was discharged with a chest tube-free drain (Fig. 4C).

## Discussion

BPF is a fatal complication of major lung surgeries, with mortality rates ranging from 27 to 71% [1]. Typically, BPFs are right-side-dominant, particularly after surgical intervention involving the intermediate bronchus [2]. The right main bronchus is predominantly supplied by a single bronchial artery, and damage to this artery can result in ischemia of the remaining bronchial stump [3]. Various risk factors have been identified, including neoadjuvant therapy, pre-operative radiotherapy, a long bronchial stump, postoperative dependence on mechanical ventilation, and stump closure technique [4–7]. Conventional surgical repair using a two-stage Clagett window or Eloesser flap following muscle flap coverage of the fistula is associated with a success rate of approximately 80% [8, 9]. Some studies suggest that bronchoscopic therapy may be an alternative treatment option to surgical repair, especially in individuals who are unsuitable for surgery [8, 10, 11]. However, most of these studies were case series or comparative studies with carefully selected patients, and there is no consensus regarding the choice of blockage material and appropriate size of the fistula for treatment.

Marwah et al. proposed a protocol to determine the size and treatment options for fistulas [12]. Fistula size may be a crucial determinant of the blockage material. Small BPFs (1–3 mm) may heal with only conservative treatment such as mechanical abrasion or endobronchial glue [13]. However, for larger fistulas (> 6 mm), an occluder or stent placement may be required. Amplatzer occluders are the most commonly used devices for the non-surgical management of BPF. Amplatzer devices are designed for atrial septal defects, patent foramen ovale, and vascular plug closure. Fruchter et al. demonstrated a success rate of 96% in a case series involving 31 patients with BPFs ranging in size from approximately 6 mm [14]. Although promising, Amplatzer devices are associated with some limitations. First, these Amplatzer devices are made of nitinol mesh and polyester materials, which have no ductility and may allow for air leakage. Second, Amplatzer devices are expensive and are not paid for by the National Health Insurances in Taiwan. Therefore, alternative blocking agents are required.

The abdominal fat pad, predominantly composed of adipocytes, adipose-derived stem cells (ASCs), and connective tissue, exhibits remarkable malleability and is cost-effective. These fat pads are rich in capillary networks and may induce neovascularization at the stump side. Autologous fat transplantation was first described in plastic surgery [15]. Although autologous fat grafting become increasingly popular, it is associated with 40–50% central necrosis. To overcome fat necrosis and fistula recurrence, we applied the PRP for improved the autologous fat growth. PRP promotes early vascularization in fat grafting, as evidenced by both in vitro and in vivo studies [14, 16–19]. In our case, we isolated two fragments of the abdominal subcutaneous fat pads, each measuring 1 × 1 cm. According to an in vitro observational study, adipose tissue can survive in severely hypoxic environments for up to 3 days, with potential degeneration of nearly 20% over 6 weeks and nearly 50% over 14 weeks. However, with PRP assistance, fat tissue survival increased to 20% volume loss at 14 weeks [20]. In our case, with the assistance of PRP, the fat pad did not shrink until Day 60 post-surgery. Fat-pad shrinkage may interfere with the success of fistula repair. Once the fat pad graft becomes necrotic and apoptotic, the bottom fistula may recur with a large volume air leak. Based on the micro-autologous fat transplantation theory proposed by Lin et al. in 2006, optimal autologous fat transplantation methods are micro-adipose particles within a radius of 1 mm [21]. However, the fluid-like material was not well-maintained in the bronchial stump. Therefore, we suggest using multiple fat tissue fragments to fill the entire fistula to avoid central necrosis of the fat pad. Marchioni et al. had another successful case report with fat pad treatment for BPF, and the fat pad retrieval sizes were approximately 1 cm [22]. In our case, the two fat pads, each measuring 1 × 1 cm, effectively filled the entire upper-lobe bronchus and successfully addressed the large BPF. Further studies are required to confirm the limitations and utility of this method.

## Data Availability

No datasets were generated or analysed during the current study.

## Abbreviations

BPF: Bronchopulmonary fistula
VSD: Ventricular septal defects
PRP: Platelet rich plasma
FEV1: Forced expiratory volume
FVC: Forced vital capacity
